# Supplementary material for: Vertical structural complexity of plant communities represents the combined effects of resource acquisition and environmental stress on the Tibetan Plateau
Source: Commun Biol. 2024 Apr 1;7:395. doi: 10.1038/s42003-024-06076-x (PMC10984992; doi:10.1038/s42003-024-06076-x)
Supplement: Supplementary file 2 — Supplementary Information [file 42003_2024_6076_MOESM2_ESM.pdf]

Vertical structural complexity of plant communities represents the combined effects of resource acquisition and environmental stress on the Tibetan Plateau

Changjin Cheng [1, 2], Jiahui Zhang[4*], Mingxu Li [3], Congcong Liu [3], Li Xu [3], Nianpeng He [4,5*]

[1] *State Key Laboratory of Plant Diversity and Specialty Crops, South China Botanical Garden, Chinese Academy of Sciences, Guangzhou 510650, China*

[2] *School of Ecology and Nature Conservation, Beijing Forestry University, Beijing 100083, China*

[3] *Key Laboratory of Ecosystem Network Observation and Modeling, Institute of Geographic Sciences and Natural Resources Research, Chinese Academy of Sciences, Beijing 100101, China*

[4] Key Laboratory of Sustainable Forest Ecosystem Management - Ministry of Education, Northeast Forestry University, Harbin, 150040, China

[5] Northeast Asia ecosystem Carbon sink research Center, Northeast Forestry University, Harbin, 150040, China

*Corresponding authors: Jiahui Zhang (e-mail: zhangjiahui@nefu.edu.cn) and Nianpeng He (e-mail: henp@igsnrr.ac.cn; Tel.: +86 10 64889263; Fax: +86 10 64889432)

SUPPORTING INFORMATION

Table S1 Literature statistics on the overyielding effect explained by vertical structural complexity.

Search number	Categories	Search terms	Number of studies
1	Resource partitioning	(‘resource complementarity’ OR ‘resource partitioning’ OR ‘niche partitioning’ OR ‘niche differentiation’) AND biodiversity AND ecosystem function* AND plant* AND complementarity	699
2	vertical structural complexity	‘vertical structural complexity’ OR ‘spatial complementarity’ OR ‘spatial partitioning’ OR ‘crown complementarity’ OR ‘physical niche partitioning’	136
3	Quantified aboveground spatial partitioning		10
	Forest		7
	Grassland		3

†We conducted a literature search based on the Web of Science. We set the search in the context of “Resource partitioning”, as vertical structural complexity is often used as a type of resource partitioning to explain overyielding effects in the framework of complementary effects theory. For the search term “Resource partitioning” we refer to Barry *et al.* (2019). For the search term “vertical structural complexity” we have aggregated similar expressions from the literature. The term “Quantified aboveground spatial partitioning” was identified mainly by reading the abstracts of the literature, and where this was not possible, we read the full text. Our search period was 1950-2022.

Table S2 Partial correlations between Height-max, Height-var and Height-even and the variable that dominates its variation in the multiple stepwise regression models. We explore the effect of sampling on the results by controlling for the effect of Shannon index. There is no significant difference between zero-order (no control for the effects of other variables) and partial correlation coefficient, indicating that the sampling method has no significant effect on the results.

	Alpine meadow			Alpine steppe			Alpine desert		
	Height -max	Height -var	Height-even	Height-max	Height-var	Height-even	Height-max	Height-var	Height-even
Zero-order (Pearson correlation)									
MAT	—	0.77	—	—	0.61	-0.21	—	—	—
$T_{diurnal}$	—	—	—	-0.7	—	—	-0.56	—	—
Wind	-0.53	-0.69	0.31	—	-0.61	—	-0.26	-0.42	0.21
PO_2	0.54	—	—	—	—	—	—	—	—
Partial correlation (control Shannon's influence)									
MAT	—	0.77	—	—	0.65	-0.22	—	—	—
$T_{diurnal}$	—	—	—	-0.7	—	—	-0.56	—	—
Wind	-0.49	-0.71	0.3	—	-0.61	—	-0.27	-0.4	0.23
PO_2	0.49	—	—	—	—	—	—	—	—

† Height-max, maximum plant height within a plot; Height-var, the coefficient of variation of plant height; Height-even, the Shannon evenness of plant height; MAT, annual mean temperature; Wind, wind speed; PO_2, atmospheric oxygen partial pressure; $T_{diurnal}$, diurnal temperature range.

Table S3 Multiple stepwise regression models (based on random sampling) of the relationship between vertical structural complexity and environmental variables, constructed using different vegetation types.

	Height-max					Height-var				
		P	VIF	R^2			*P*	VIF	R^2	
	AI	<0.001	1.086	0.465		AI	<0.001	1.217	0.368	
Forest	Wind	0.165	2.169	0.034	0.512	MAT	0.002	1.207	0.122	0.541
	MAT	0.269	2.148	0.012		pH	0.587	1.151	0.035	
						UR	0.719	1.154	0.017	
		P	VIF	R^2			*P*	VIF	R^2	
	$T_{diurnal}$	0.001	1.311	0.107		MAT	<0.001	4.21	0.316	
	PO_2	0.012	2.413	0.098		Wind	0.195	4.991	0.253	0.737
Alpine meadow	Wind	0.022	4.187	0.086	0.378	PO_2	0.412	2.316	0.106	
	MAT	0.172	4.234	0.07		AI	0.374	1.493	0.062	
	AI	0.21	2.099	0.012						
	pH	0.676	1.504	0.004						
		P	VIF	R^2			*P*	VIF	R^2	
	$T_{diurnal}$	<0.001	1.24	0.51		Wind	<0.001	3.721	0.229	
Alpine steppe	Wind	0.017	3.194	0.032	0.594	MAT	0.001	2.8	0.205	
	AI	0.875	1.36	0.028		PO_2	0.011	2.897	0.079	0.54
	MAT	0.791	2.9	0.024		SOC	0.052	1..163	0.017	
						AI	0.263	1.328	0.009	
		P	VIF	R^2			*P*	VIF	R^2	
	$T_{diurnal}$	<0.001	1.362	0.205		Wind	<0.001	1.893	0.174	
Alpine desert	Wind	0.008	1.168	0.11	0.33	UR	<0.001	1.534	0.151	0.445
	AI	0.138	1.288	0.015		$T_{diurnal}$	0.007	1.562	0.091	
						MAT	0.079	2.175	0.029	

† All models are significant to $p < 0.001$. R^2 for each model and its independent variables is provided separately. Height-max, maximum plant height within a plot; Height-var, the coefficient of variation of plant height; VIF, variance inflation factor; MAT, annual mean temperature; AI, aridity index; Wind, wind speed; PO_2, atmospheric oxygen partial pressure; UR, ultraviolet radiation; $T_{diurnal}$, diurnal temperature range; SOC, soil organic carbon; TN, soil nitrogen content.

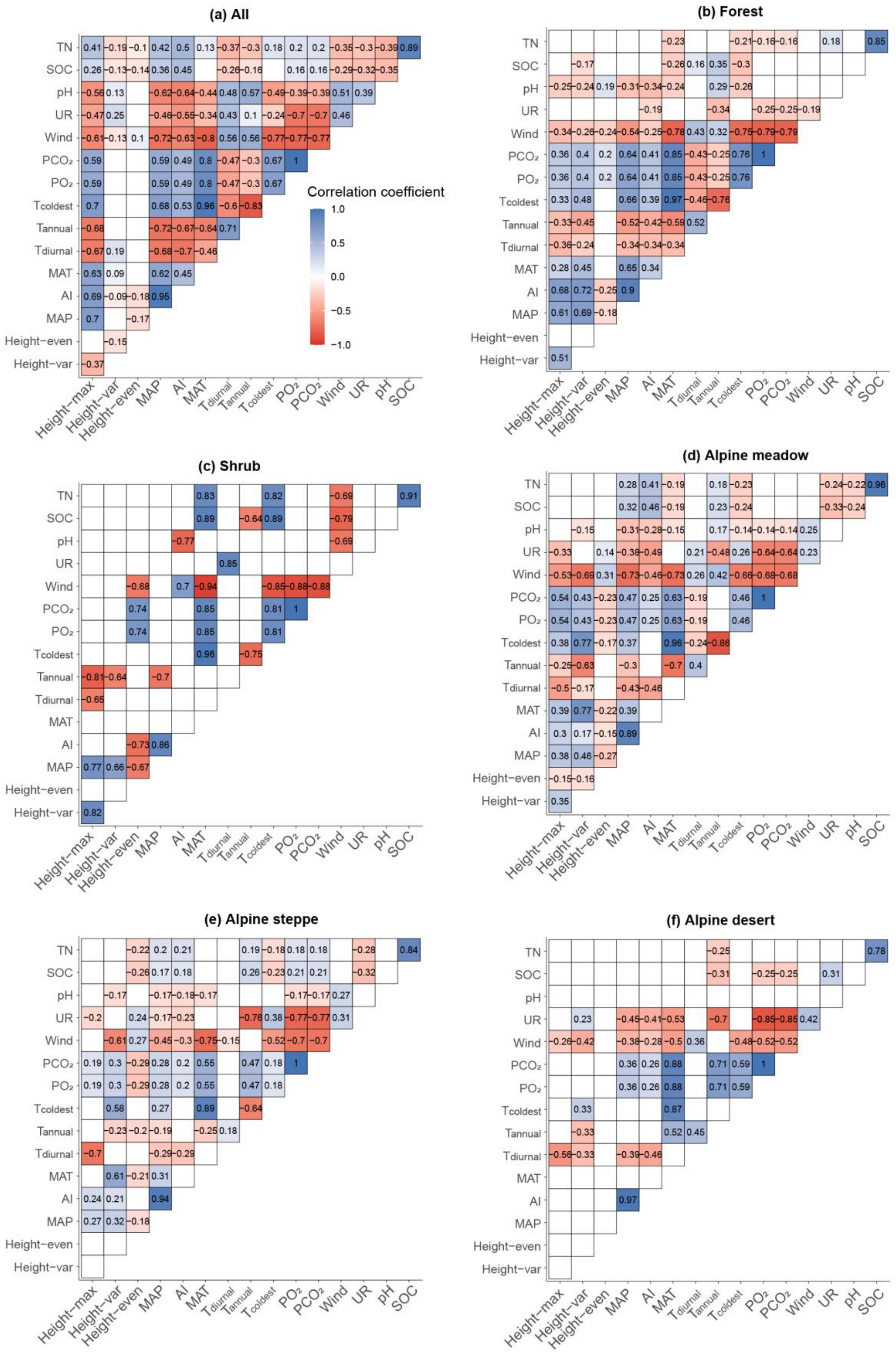

Figure S1 Pearson correlation between variables. Blank means not significant at p = 0.05 level. All, across all sites; Height-max, maximum plant height within a plot;

Height- var, the variation coefficient of plant height; Height-even, the Shannon evenness of plant height; AI, aridity index; MAP, annual mean temperature; MAT, annual mean temperature; $T_{diurnal}$, diurnal temperature range; T_{annual}, temperature annual range; $T_{coldest}$, min temperature of coldest month; Wind, wind speed; UR, ultraviolet radiation; PO_2, atmospheric oxygen partial pressure; PCO_2, atmospheric carbon dioxide partial pressure; TN, soil nitrogen content; SOC, soil organic carbon.

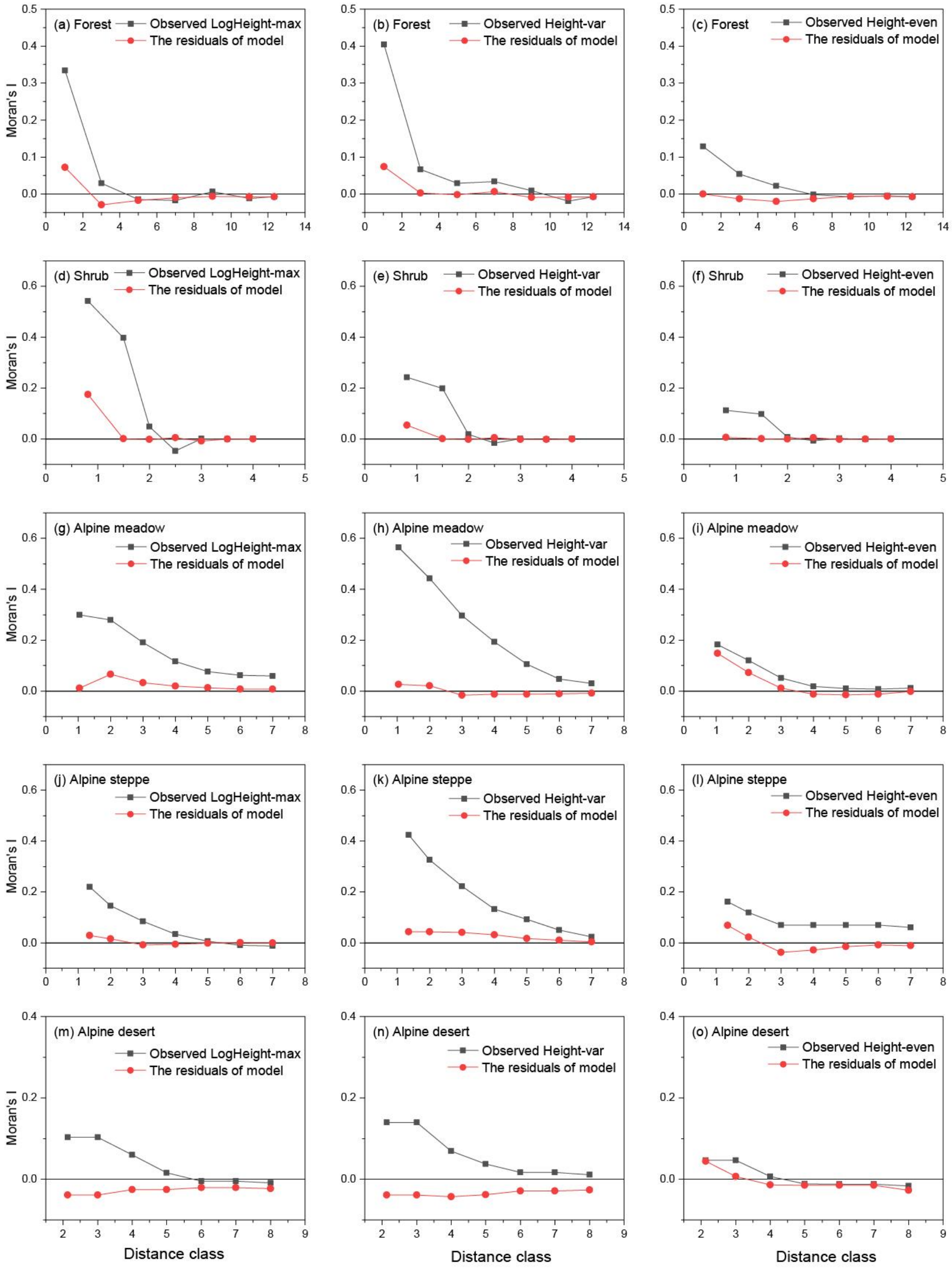

Figure S2 Spatial correlograms for the observations and the residuals of the corresponding multivariate stepwise regression model in Table 2. Spatial correlograms are estimated by Moran's I coefficients. In the figures, black squares represent Moran's I for observations of LogHeight-max, Height-var and Height-even, while red circles represent Moran's I for the residuals. Height-max, maximum plant height within a plot; Height-var, the variation coefficient of plant height; Height-even, the Shannon evenness of plant height.

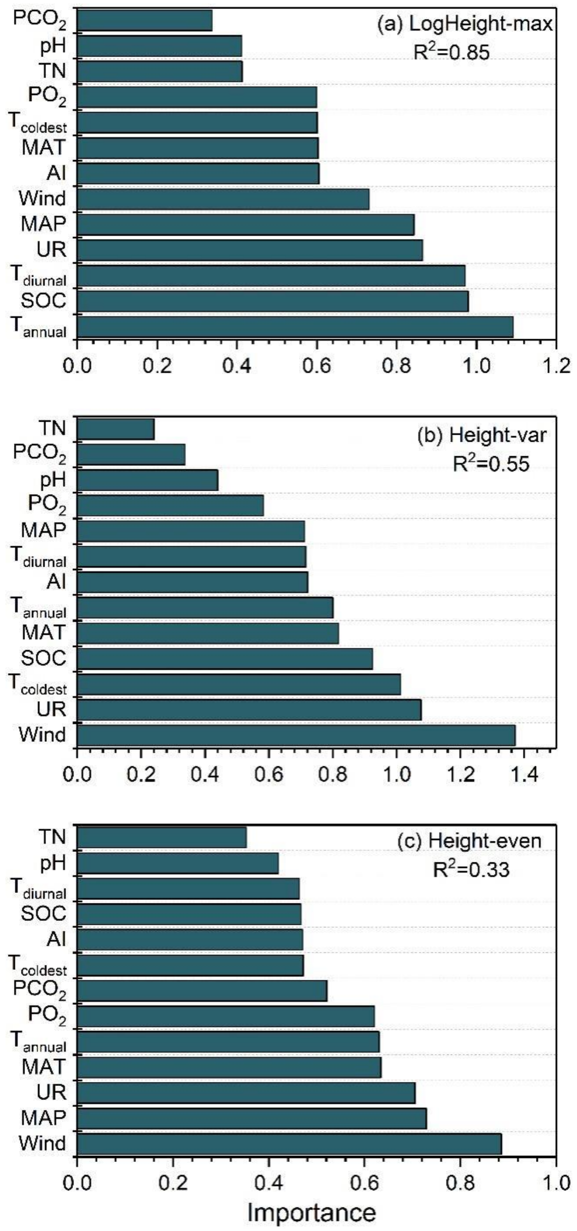

Figure S3 Importance of the explanatory variables for the spatial variation of vertical structural complexity on the Tibetan Plateau. The increase in node purity of the splitting variables was used to estimate the relative importance of the predictors. Height-max, maximum plant height within a plot; Height-var, the coefficient of variation of plant height; Height-even, the Shannon evenness of plant height; AI, aridity index; MAP, annual mean temperature; MAT, annual mean temperature; Tdiurnal, diurnal temperature range; Tannual, temperature annual range; Tcoldest, min temperature of coldest month; Wind, wind speed; UR, ultraviolet radiation; PO_2, atmospheric oxygen partial pressure; PCO_2, atmospheric carbon dioxide partial pressure; TN, soil nitrogen content; SOC, soil organic carbon.

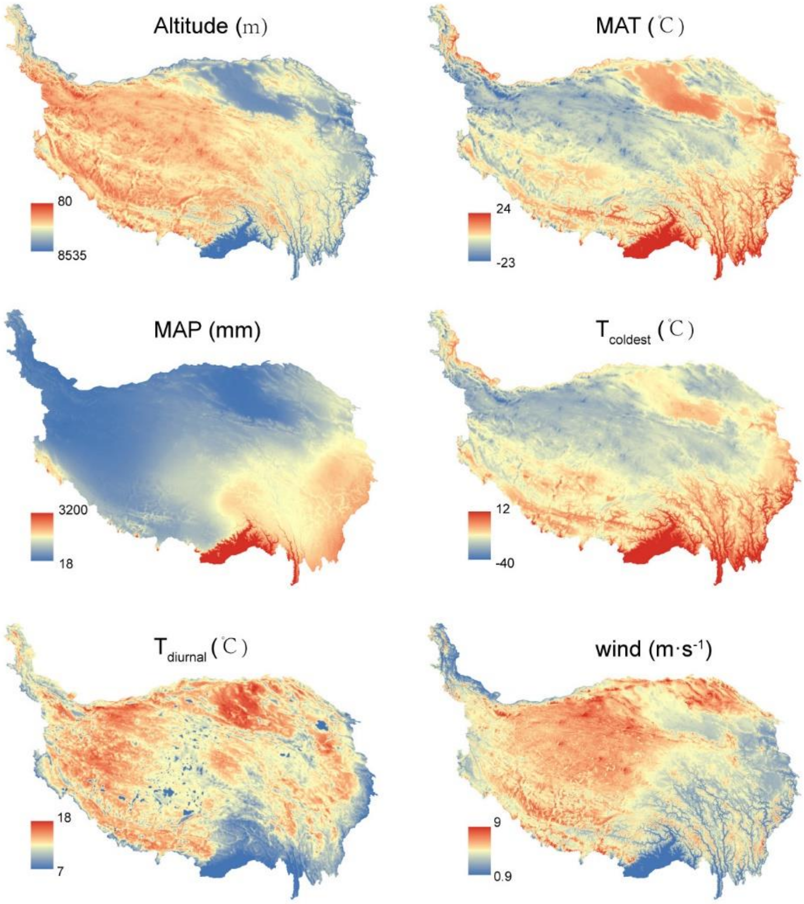

Figure S4 Spatial pattern of altitude and climatic factors in the Tibetan Plateau. The climate factor comes from WorldClim, and the altitude comes from Resource and Environment Science and Data Center. MAT, annual mean temperature; MAP, annual mean temperature; $T_{coldest}$, min temperature of coldest month; $T_{diurnal}$, diurnal temperature range; Wind, wind speed.

References

Barry, K.E. *et al.* The Future of Complementarity: Disentangling Causes from Consequences. *Trends Ecol. Evol.* **34**, 167-180 (2019).

Lefsky, M. A. A global forest canopy height map from the Moderate Resolution Imaging Spectroradiometer and the Geoscience Laser Altimeter System. Geophys. Res. Lett. **37**, L15401 (2010).
