# Supplementary material for: Vertical structural complexity of plant communities represents the combined effects of resource acquisition and environmental stress on the Tibetan Plateau
Source: Commun Biol. 2024 Apr 1;7:395. doi: 10.1038/s42003-024-06076-x (PMC10984992; doi:10.1038/s42003-024-06076-x)
Supplement: Supplementary file 3 — Description of Additional Supplementary Files [file 42003_2024_6076_MOESM3_ESM.pdf]

Description of Additional Supplementary Files

File name: Supplementary Data

Description: All data for the figures and tables in the article
