# Supplementary material for: Vertical structural complexity of plant communities represents the combined effects of resource acquisition and environmental stress on the Tibetan Plateau
Source: Commun Biol. 2024 Apr 1;7:395. doi: 10.1038/s42003-024-06076-x (PMC10984992; doi:10.1038/s42003-024-06076-x)
Supplement: Supplementary file 5 — Reporting Summary [file 42003_2024_6076_MOESM5_ESM.pdf]

nature portfolio

Corresponding author(s): Nianpeng He

Last updated by author(s): Mar 18, 2024

Reporting Summary

Nature Portfolio wishes to improve the reproducibility of the work that we publish. This form provides structure for consistency and transparency in reporting. For further information on Nature Portfolio policies, see our Editorial Policies and the Editorial Policy Checklist.

Statistics

For all statistical analyses, confirm that the following items are present in the figure legend, table legend, main text, or Methods section.

n/a	Confirmed	
☐	☒	The exact sample size (*n*) for each experimental group/condition, given as a discrete number and unit of measurement
☐	☒	A statement on whether measurements were taken from distinct samples or whether the same sample was measured repeatedly
☐	☒	The statistical test(s) used AND whether they are one- or two-sided<br>*Only common tests should be described solely by name; describe more complex techniques in the Methods section.*
☐	☒	A description of all covariates tested
☐	☒	A description of any assumptions or corrections, such as tests of normality and adjustment for multiple comparisons
☐	☒	A full description of the statistical parameters including central tendency (e.g. means) or other basic estimates (e.g. regression coefficient) AND variation (e.g. standard deviation) or associated estimates of uncertainty (e.g. confidence intervals)
☐	☒	For null hypothesis testing, the test statistic (e.g. *F*, *t*, *r*) with confidence intervals, effect sizes, degrees of freedom and *P* value noted<br>*Give P values as exact values whenever suitable.*
☒	☐	For Bayesian analysis, information on the choice of priors and Markov chain Monte Carlo settings
☒	☐	For hierarchical and complex designs, identification of the appropriate level for tests and full reporting of outcomes
☒	☐	Estimates of effect sizes (e.g. Cohen's *d*, Pearson's *r*), indicating how they were calculated

Our web collection on statistics for biologists contains articles on many of the points above.

Software and code

Policy information about availability of computer code

Data collection	The height data primarily used in this study were obtained from standardized field surveys. More details are reported in the Methods section.
Data analysis	All statistical analyses were performed using the R statistical software package (ver. 4.1.0). Details were reported in the Method section about Statistical analysis.

For manuscripts utilizing custom algorithms or software that are central to the research but not yet described in published literature, software must be made available to editors and reviewers. We strongly encourage code deposition in a community repository (e.g. GitHub). See the Nature Portfolio guidelines for submitting code & software for further information.

Data

Policy information about availability of data

All manuscripts must include a data availability statement. This statement should provide the following information, where applicable:

- Accession codes, unique identifiers, or web links for publicly available datasets
- A description of any restrictions on data availability
- For clinical datasets or third party data, please ensure that the statement adheres to our policy

All data for the figures and tables in the article are provided in the Supplementary Data.

Research involving human participants, their data, or biological material

Policy information about studies with human participants or human data. See also policy information about sex, gender (identity/presentation), and sexual orientation and race, ethnicity and racism.

Reporting on sex and gender	N/A
Reporting on race, ethnicity, or other socially relevant groupings	N/A
Population characteristics	N/A
Recruitment	N/A
Ethics oversight	N/A

Note that full information on the approval of the study protocol must also be provided in the manuscript.

Field-specific reporting

Please select the one below that is the best fit for your research. If you are not sure, read the appropriate sections before making your selection.

☐ Life sciences ☐ Behavioural & social sciences ☒ Ecological, evolutionary & environmental sciences

For a reference copy of the document with all sections, see nature.com/documents/nr-reporting-summary-flat.pdf

Ecological, evolutionary & environmental sciences study design

All studies must disclose on these points even when the disclosure is negative.

Study description	Empirical research
Research sample	We systematically investigated 2,013 plant communities through grid sampling on the Tibetan Plateau.
Sampling strategy	Plant samples were collected using the quadrat method (20 m × 20 m for the forest and shrub and 1 m × 1 m for grassland) to investigate the community structure during the high-growth period from mid-July to late August in 2018, 2019, and 2020.
Data collection	In each plot within a site, key plant community structure variables were measured.
Timing and spatial scale	Field surveys were conducted from 2018-2020 and the surveyed sites extended from 25°N to 40°N and 74°E to 104°E.
Data exclusions	No outliers were found.
Reproducibility	Standardized sampling and measurement protocols were applied to each site's vegetation and soil surveys.
Randomization	Three tree and grassland plots within a site were randomly set and to reduce soil heterogeneity, we combined the soil samples collected randomly within each plot into a composite sample. See the Methods section for more details.
Blinding	N/A

Did the study involve field work? ☒ Yes ☐ No

Field work, collection and transport

Field conditions	Mean annual temperatures (MAT) ranging from −23°C to 24°C and mean annual precipitation (MAP) ranging from 18–3200 mm for these studied sites.
Location	The surveyed sites extended from 25°N to 40°N and 74°E to 104°E.
Access & import/export	This work was supported by the the second Tibetan Plateau Scientific Expedition and Research Program.
Disturbance	The plots were located far away from the main human disturbances according to standard protocols for sampling.

Reporting for specific materials, systems and methods

We require information from authors about some types of materials, experimental systems and methods used in many studies. Here, indicate whether each material, system or method listed is relevant to your study. If you are not sure if a list item applies to your research, read the appropriate section before selecting a response.

Materials & experimental systems

n/a	Involved in the study
☒	☐ Antibodies
☒	☐ Eukaryotic cell lines
☒	☐ Palaeontology and archaeology
☒	☐ Animals and other organisms
☒	☐ Clinical data
☒	☐ Dual use research of concern
☒	☐ Plants

Methods

n/a	Involved in the study
☒	☐ ChIP-seq
☒	☐ Flow cytometry
☒	☐ MRI-based neuroimaging

Plants

Seed stocks	N/A
Novel plant genotypes	N/A
Authentication	N/A
